# Health risk assessment of heavy metals in black tea infusion by Monte Carlo simulation

**DOI:** 10.1002/fsn3.4452

**Published:** 2024-10-21

**Authors:** Fatemeh Esfarjani, Mohammad Rouzbahani, Seyed Ehsan Beladian Behbahan, Narges Shahbazpour, Masoumeh Moslemi, Abdol‐Samad Abedi

**Affiliations:** ^1^ Food and Nutrition Policy and Planning Research Department, National Nutrition and Food Technology Research Institute (NNFTRI), Faculty of Nutrition Sciences and Food Technology Shahid Beheshti University of Medical Sciences Tehran Iran; ^2^ National Nutrition and Food Technology Research Institute (NNFTRI), Faculty of Nutrition Sciences and Food Technology Shahid Beheshti University of Medical Sciences Tehran Iran; ^3^ Community Medicine Department, School of Medicine Shahid Beheshti University of Medical Sciences Tehran Iran; ^4^ Halal Research Center of IRI., Iran Food and Drug Administration, Ministry of Health and Medical Education Tehran Iran

**Keywords:** heavy metal, Monte Carlo, risk assessment, tea infusion, tea leave

## Abstract

Tea leaves and their infusion have been interested in the populations because of their therapeutic and relaxing effects. However, tea plant is prone to heavy metals' bioaccumulation. Regarding the high consumption of tea infusion, concentration of arsenic, cadmium, and lead in black tea infusion was determined by inductively coupled plasma mass spectrometry. Tea infusion was prepared by addition of 45 mL deionized water to 1 g tea leaves followed by heating at 90°C for 10 min. After analysis, carcinogenic and noncarcinogenic risks of heavy metals were investigated by Monte Carlo simulation in two age groups of children and adults younger and older than 15 years old, respectively. According to the results, Hazard Quotient (HQ) of three heavy metals in both children and adults was equal and lower than 0.01. Furthermore, incremental lifetime cancer risk (ILCR) of arsenic in Iranian black tea consumers (<1.5 × 10^−6^) and lead in all consumers (about 2.55 × 10^−7^) was within the acceptable range introduced by US EPA (≤10^−6^). ILCR of arsenic in children and adults consuming foreign tea infusion was within the range of 10^−6^–10^−4^. Our further investigation revealed that the highest area (75%–85%) of cumulative frequency distribution for arsenic ILCR in foreign tea consumers was related to ILCR ≤10^−5^ which is the acceptable range determined by the World Health Organization. Therefore, there was no serious concern about the intake of heavy metals by tea infusion in Iranian children and adults.

## Graphical abstract

Regarding the high consumption of tea infusion, concentrations of arsenic, cadmium, and lead in Iranian and foreign black tea infusions were determined by inductively coupled plasma mass spectrometry. We concluded that there was no serious concern about the intake of heavy metals by tea infusion in Iranian children and adults.

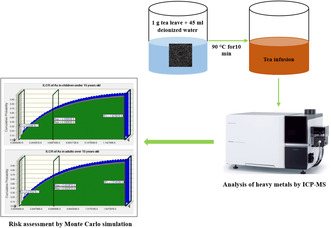

## 1 INTRODUCTION

Tea is produced from the leaves of the *Camellia sinensis* plant in the family Theaceae. Black and green tea are the most popular types of tea in the world. Black tea is prepared after the fermentation process through which a black color is formed by chemical reactions developed by oxidizing enzymes (Mahmood et al., 2010). Due to its beneficial role in the body such as antioxidant activity, anticarcinogenic effect, and prevention of some noncommunicable diseases (cardiovascular diseases and diabetes), tea is of interest in traditional medicine (Karami et al., 2019; Shin et al., 2022; Wang et al., 2021). According to the report of the Food and Agriculture Organization, global production and consumption of tea increased from approximately 3,500,000 tons to more than 6,000,000 tons from 2005 to 2020. It is largely produced by Asian countries including India, China, and Turkey. Iran is the sixth tea‐consuming country in the world (Food and Agriculture Organization of the United Nations, 2022). It is estimated that 3–3.5 g/day of black tea is consumed in Iran (Karimi et al., 2008; Parviz et al., 2015).

Tea leaves are prone to heavy metal accumulation. Concentration of heavy metals in the plant is determined by genetics (Wei et al., 2023) and environmental factors such as soil contamination, pH, and geography (Girolametti et al., 2023). These contaminants change the plants' metabolism and affect the nutrient content in tea leaves (Peng et al., 2023). In addition, long‐term exposure to heavy metals is dangerous to humans and may pose the body at risk of different cancers and other health‐threatening conditions. For example, arsenic interacts with thiol groups in the body and interferes with the phosphorylation process. Therefore, moderate to high intake of arsenic is associated with oxidative stress, inflammation, dyslipidemia, glucose metabolism disorder, cardiovascular diseases, and stroke (Karachaliou et al., 2022; Khan et al., 2022). Other than possible incidence of cardiovascular diseases and osteoporosis (Bimonte et al., 2021), development of renal diseases has been addressed after chronic exposure to cadmium (Nishijo et al., 2020). Similar to arsenic and cadmium, lead exposure induces oxidative stress and immune response in the body (Ebrahimi et al., 2020). It has been reported that lead exposure contributes to 500,000 deaths and 9.3 million years of life loss among adults 15 years or older (Glicklich & Frishman, 2021). In this regard, a tolerable intake as a reference dose has been determined by international health agencies for these contaminants to minimize the risk of associated diseases in populations. Among all routes of intake in humans, edible intake of the contaminants is of great importance. Considering the high consumption of tea in the world and especially in Iran, evaluation of its role in the prevalence of cancer and noncarcinogenic diseases in people is important. Therefore, we aimed to develop a risk assessment study on three heavy metals (arsenic, cadmium, and lead) delivered by black tea infusion in children and adults younger and older than 15 years old, respectively. For this, both Iranian black tea and imported black tea to Iran were studied.

## 2 MATERIALS AND METHODS

### 2.1 Samples

Different types of black tea including broken orang pekoe, orang pekoe, CTC (crush, tear, curl), and tea bag were purchased from Tehran market (Iran). In total, 23 packaged black teas (16 foreign and 7 Iranian black teas) from different brands were analyzed.

### 2.2 Sample preparation

Tea infusion was prepared according to the method described by Naghipour et al. with some modifications (Naghipour et al., 2016). At first, 45 mL of deionized water was added to 1 g of tea leaves, and the mixture was heated at 90°C for 10 min. Then, the mixture was filtered by Whatman paper (2.5 μm). In the next step, 3 mL perchloric acid and 2 mL hydrogen peroxide were added to the filtrate, and the solution was made up to 50 mL by deionized water. The final solution was analyzed by inductively coupled plasma mass spectrometry (ICP‐MS, ULTIMA2, 6100 DRC‐e Perkin Elmer Elan) to determine the concentration of arsenic, cadmium, and lead. The same method was used for preparation of blank without addition of tea leaves. ICP‐MS was adjusted according to the condition described by Abedi et al. (2023). Details of ICP‐MS are shown in Table 1. All chemicals and standard solutions were purchased from Merck (Germany).

**TABLE 1:** Conditions of ICP‐MS apparatus for determination of arsenic, cadmium, and lead.

Parameter	Value
Radiofrequency	1200 W (40 Mhz)
Plasma gas (Argon) flow	16 L/min
Nebulizer gas (Argon) flow	1 L/min
Read delay and analysis speeding	30 s
Wash	60 s
Wash speeding	30 rpm
Dwell time	50 ms
Resulting/amu 10% peak	0.7
Integration time	3.5
Repetition	3
Linear working standard (μg/l)	5–100
*R* ^2^	91–99
RSD% (*n* = 10)	1.3–2
Addition/recovery	91–93
Limit of detection (LOD) (μg/kg)	0.0003
Limit of quantification (LOQ) (μg/kg)	0.001

### 2.3 Risk assessment

To calculate the risks arising from the hazards (i.e., heavy metals in tea infusion), average body weight of individuals in the population was required. In this study, two groups of individuals including children and adults younger and older than 15 years were investigated. The average body weight was considered 50 and 65 kg for children and adults, respectively. In the next step, average daily consumption of tea infusion by individuals was estimated according to the reports of Islami et al. (Islami et al., 2009) and Rezaee et al. (Rezaee et al., 2016) in Iran. Concentration of arsenic, cadmium, and lead in tea infusion was determined in the laboratory (Abedi et al., 2023; Hoseini et al., 2023). Tolerable daily intake (TDI) and carcinogenic slope factor (CSF) of the heavy metals were extracted from the reports of international health agencies.

#### 2.3.1 Noncarcinogenic risk

Daily intake of each heavy metal through consumption of tea infusion was estimated according to Equation 1. Then, Hazard Quotient (HQ) was calculated according to Equation 2 to determine noncarcinogenic risk arising from arsenic, cadmium, and lead by consumption of tea infusion in both groups of children and adults (Abedi et al., 2023; Hoseini et al., 2023).

(1) $$\text{Estimated daily intake}\;\left(\mathrm{EDI}\right)=\frac{\;\mathrm{DC}\;\times C_{m}\;}{\mathrm{BW}}$$

where DC is the daily consumption of tea infusion (L/day), C_m_ is the average concentration of each heavy metal in tea infusion (μg/L), and BW is body weight (kg).

(2) $$\mathrm{HQ}=\frac{\mathrm{EDI}}{\mathrm{RfD}}$$

where RfD is the reference dose determined for each heavy metal by international health agencies, that is, 3 × 10^−4^ mg/kg bw day for arsenic (US Environmental Protection Agency, 1991), 1 × 10^−3^ mg/kg bw day for cadmium (US Environmental Protection Agency, 1989), and 0.004 mg/kg bw day for lead (Nag & Cummins, 2022).

Hazard index (HI), that is, sum of HQs, was calculated according to Equation 3.

(3) $$\mathrm{HI}=\sum_{i}^{n}{\mathrm{HQ}}_{s}$$

#### 2.3.2 Carcinogenic risk

Incremental lifetime cancer risk (ILCR) formula (Equation 4) was used for the estimation of carcinogenic risk in the individuals consuming tea infusion. A safe limit of lower than 10^−6^ ILCR (i.e., <1 in 1,000,000 persons) and a critical limit of more than 10^−4^ ILCR (i.e., >1 in 10,000 persons) have been determined by the United States Environmental Protection Agency (US EPA). In Monte Carlo simulation, as a probabilistic risk assessment approach, the decision on ILCR between 10^−6^ and 10^−4^ depends on the location of percentiles in the risk distribution graph. On the other hand, it is important to find which percentiles in risk distribution are within or out of the range (US Environmental Protection Agency, 2001). To help better decision by the risk managers, cut‐off point of 10^−5^ has been introduced for ILCR by the World Health Organization (Bostrom et al., 2002; Gong et al., 2017).

(4) ILCR=EDI×CSF

where CSF is the carcinogenic slope factor of each heavy metal determined by international health agencies. CSF is 1.5 mg/kg bw day for arsenic (US Environmental Protection Agency, 1991) and 0.0085 mg/kg bw day for lead (OEHHA, 2023). No CSF was introduced for cadmium.

### 2.4 Statistical analysis

Data of heavy metal contamination in tea leaves and infusion were analyzed by Excel software version 2019. They are presented as mean ± standard deviation (Tables 2, 3, 4). To reduce the uncertainties in the estimation of the risks, Monte Carlo simulation was used as a probabilistic method. Distribution of the risks was provided by Oracle, Inc.'s Crystal Ball software (version 11.1, USA). Probability and sensitivity graphs of HI in both groups of children and adults were also drawn by Oracle Crystal Ball. The simulation was done by 10,000 iterations.

**TABLE 2:** Concentration of heavy metals in foreign black tea leaves and infusion.

Sample	Arsenic			Cadmium			Lead		
	Leaves (μg/kg)	Infusion (μg/L)	Transfer (%)	Leaves (μg/kg)	Infusion (μg/L)	Transfer (%)	Leaves (μg/kg)	Infusion (μg/L)	Transfer (%)
1	2.1	0.57	27.14	3.1	1.11	35.81	5.53	3.45	62.39
2	1.9	0.11	5.79	3.2	1.45	45.31	4.17	3.37	80.81
3	2.1	0.22	10.48	4.4	0.12	2.73	4.11	3.08	74.94
4	1.02	0.02	1.96	3.3	0.02	0.61	4.56	3.63	79.60
5	1.02	0.02	1.96	3.8	0.05	1.32	6.14	3.15	51.30
6	1.03	0.03	2.91	3.6	0.08	2.22	4.32	2.29	53.01
7	1.39	0.39	28.06	4.3	0.07	1.63	4.23	3.34	78.96
8	1.06	0.06	5.66	4.5	0.08	1.78	4.21	2.42	57.48
9	1.09	0.09	8.26	2.9	0.11	3.79	4.21	2.11	50.12
10	1.11	0.11	9.91	4.3	0.09	2.09	4.37	3.32	75.97
11	1.34	0.34	25.37	3.5	0.03	0.86	7.34	6.02	82.02
12	1.44	0.44	30.56	3.3	0.02	0.61	4.49	1.42	31.63
13	1.3	0.3	23.08	2.4	0.06	2.50	5.68	2.56	45.07
14	1.52	0.52	34.21	3.4	0.11	3.23	3.67	1.86	50.68
15	1.48	0.48	32.43	3.3	0.09	2.73	5.32	1.42	26.69
16	1.05	0.05	4.76	3.6	1.06	29.44	4.81	1.91	39.71

## 3 RESULTS AND DISCUSSION

Tea is one of the most widely consumed beverages worldwide. It is of interest in traditional medicine due to its beneficial effects on the body. Tea leaves are full of potent antioxidants including catechins, caffeine, theaflavin, gallic acid, chlorogenic acid, ellagic acid, and kaempferol‐3‐O‐glucoside (Tang et al., 2019). They are hydrogen atom or electron donors in chemical reactions and can cease the oxidation process directed by reactive oxygen species (Truong & Jeong, 2021). Tea antioxidants, especially catechins, have been introduced as natural preservatives in domestic and industrial foods. Interestingly, the antioxidant activity of tea was superior to BHT as synthetic antioxidants in meat products (Beya et al., 2021; Homayouni Rad et al., 2022). Other than its therapeutic and beneficial roles (Forouzanfar, 2011; Rady et al., 2018; Wang et al., 2020), caffeine has revitalizing and rejuvenating role in tea consumers (Xi, 2022). Nonetheless, tea plants are susceptible to environmental contamination. Heavy metals are environmental pollutants which have several adverse effects on humans. They are prone to bioaccumulation and may exist actively in vital systems for a long time (Briffa et al., 2020). Other than the oral route, heavy metals may enter the human body by inhalation and dermal route. Several heavy metals are naturally present on the earth, and some of them are cross‐contamination made by industrial processes. Because of the approved harmful effects of heavy metals in the body, their daily intake via different routes should be monitored. In this study, oral intake of three heavy metals of arsenic, cadmium, and lead through consumption of tea infusion was investigated. Considering the popularity of tea in each age group, investigation of its toxicity is of great importance.

Several factors determine the possibility of tea plant contamination by heavy metals. For example, contamination of air, soil, and irrigation water, climate change, physicochemical characteristics of soil, genetics of plants, and post‐harvest processing are significant parameters in heavy metal contamination of tea plants (Atasoy et al., 2019; Hayat et al., 2015). Heavy metals migrate from tea leaves to infusion at different rates. Lee et al. (2019) showed that the migration rate of cadmium, lead, and arsenic from leaves to infusion in black tea was higher than in green tea. The authors believe that heavy metal chelates in tea leaves are oxidized during the fermentation process in black tea preparation. Therefore, more heavy metals are transferred from black tea leaves to infusion. Moreover, infusion time and temperature of water are significant factors in the transfer rate of heavy metals. In a study by Lee et al., extending the infusion time led to more migration of heavy metals. In comparison, most of the heavy metals in tea leaves did not transfer to tea infusion in our study. As seen in Tables 2 and 3, the transfer rate of three heavy metals in both Iranian and foreign tea infusion was low. This might be due to the short infusion time (10 min) used in our study. Furthermore, it seems that the transfer rate is species‐specific, so the highest migration was observed for lead, and the transfer rate of arsenic and cadmium in foreign black tea infusion was more than in Iranian black tea infusion.

**TABLE 3:** Concentration of heavy metals in Iranian black tea leaves and infusion.

Sample	Arsenic			Cadmium			Lead		
	Leaves (μg/kg)	Infusion (μg/L)	Transfer (%)	Leaves (μg/kg)	Infusion (μg/L)	Transfer (%)	Leaves (μg/kg)	Infusion (μg/L)	Transfer (%)
1	0.3	0.02	6.67	3.3	0.17	5.15	5.22	1.94	37.16
2	0.75	0.08	10.67	2.5	0.28	11.20	5.23	5	95.60
3	0.45	0.04	8.89	3.4	0.25	7.35	5.45	3.37	61.83
4	1.6	0.6	37.50	3.8	0.2	5.26	5.46	2.6	47.62
5	0.3	0.04	13.33	3.8	0.3	7.90	4.56	4	87.72
6	0.5	0.06	12	4.3	0.4	9.30	4.6	3.3	71.74
7	0	0	0	4.8	0.4	8.33	4	3.45	86.25

**TABLE 4:** Average concentration of heavy metals in black tea leaves and infusion.

Heavy metal	Sample	Iranian black tea	Foreign black tea	Total
Arsenic	Leaves	0.56 ± 0.51	1.37 ± 0.37	0.97 ± 0.57
	Infusion	0.12 ± 0.21	0.23 ± 0.20	0.18 ± 0.08
Cadmium	Leaves	3.70 ± 0.74	3.56 ± 0.58	3.63 ± 0.10
	Infusion	0.29 ± 0.09	0.28 ± 0.47	0.29 ± 0.01
Lead	Leaves	4.93 ± 0.55	4.82 ± 0.95	4.88 ± 0.08
	Infusion	3.38 ± 0.98	2.83 ± 1.12	3.11 ± 0.39

**TABLE 5:** Carcinogenic and noncarcinogenic risks arising from heavy metals in black tea infusion (calculated by Monte Carlo simulation).

		Arsenic	Cadmium	Lead
Foreign black tea				
Estimated daily intake (μg/kg bw day)	Under 15 years	0.03	0.01	0.04
	Over 15 years	0.05	0.01	0.05
Carcinogenic risk (ILCR) (mg/kg bw day)	Under 15 years	4.5 × 10^−5^	–	3.4 × 10^−7^
	Over 15 years	7.5 × 10^−5^	–	4.25 × 10^−7^
Noncarcinogenic risk (HQ)	Under 15 years	0.01	0.01	0.01
	Over 15 years	0.01	0.01	0.01
Iranian black tea				
Estimated daily intake (μg/kg bw day)	Under 15 years	<0.001	<0.001	0.02
	Over 15 years	<0.001	<0.001	0.04
Carcinogenic risk (ILCR) (mg/kg bw day)	Under 15 years	<1.5 × 10^−6^	–	1.7 × 10^−7^
	Over 15 years	<1.5 × 10^−6^	–	3.4 × 10^−7^
Noncarcinogenic risk (HQ)	Under 15 years	<0.001	<0.001	0.01
	Over 15 years	<0.001	<0.001	0.01

**FIGURE 1:**
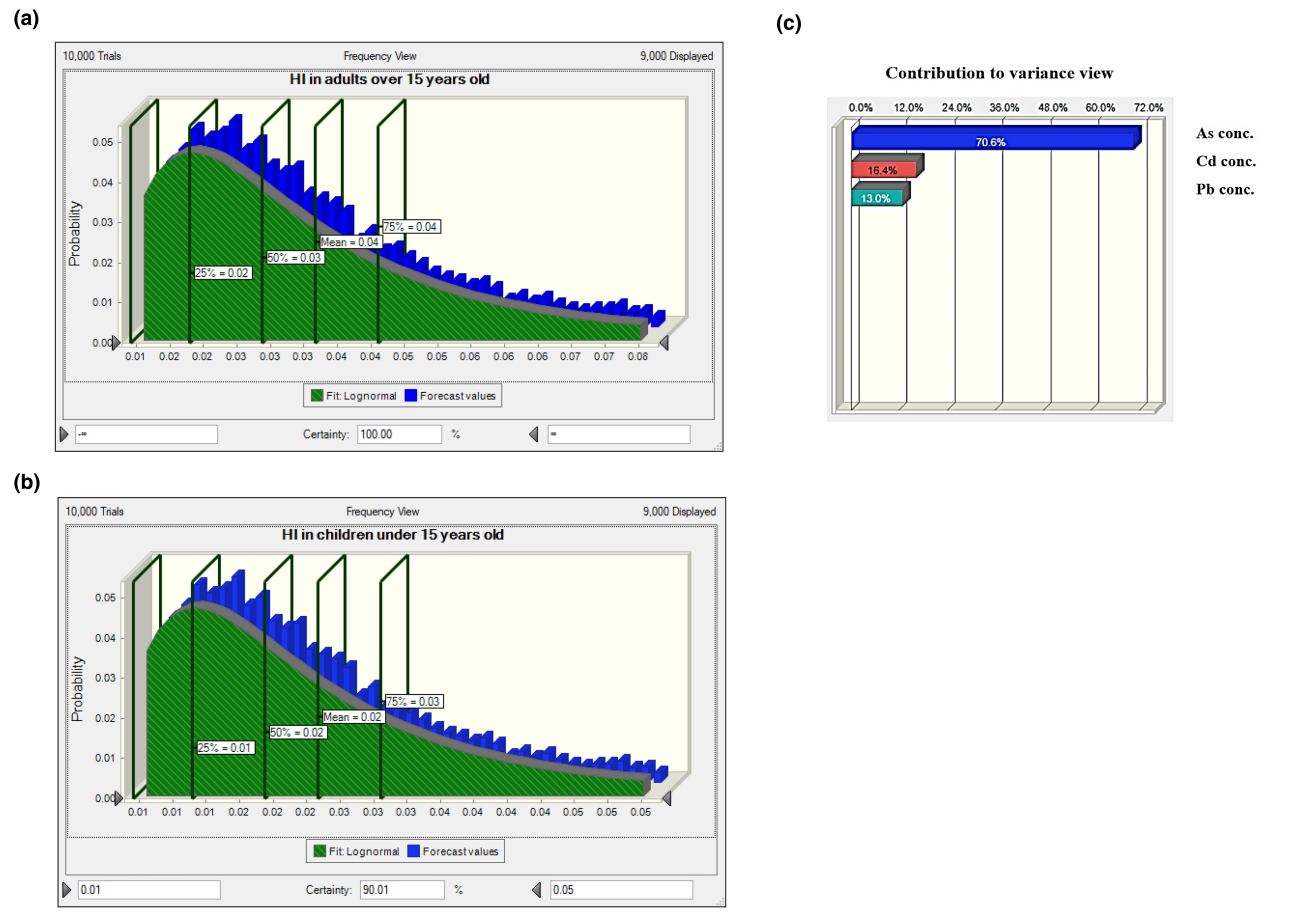
Histogram of Hazard index (HI) for heavy metals ingested by foreign black tea infusion: (a) in adults, (b) in children, (c) sensitivity chart of HI histogram in both groups.

**FIGURE 2:**
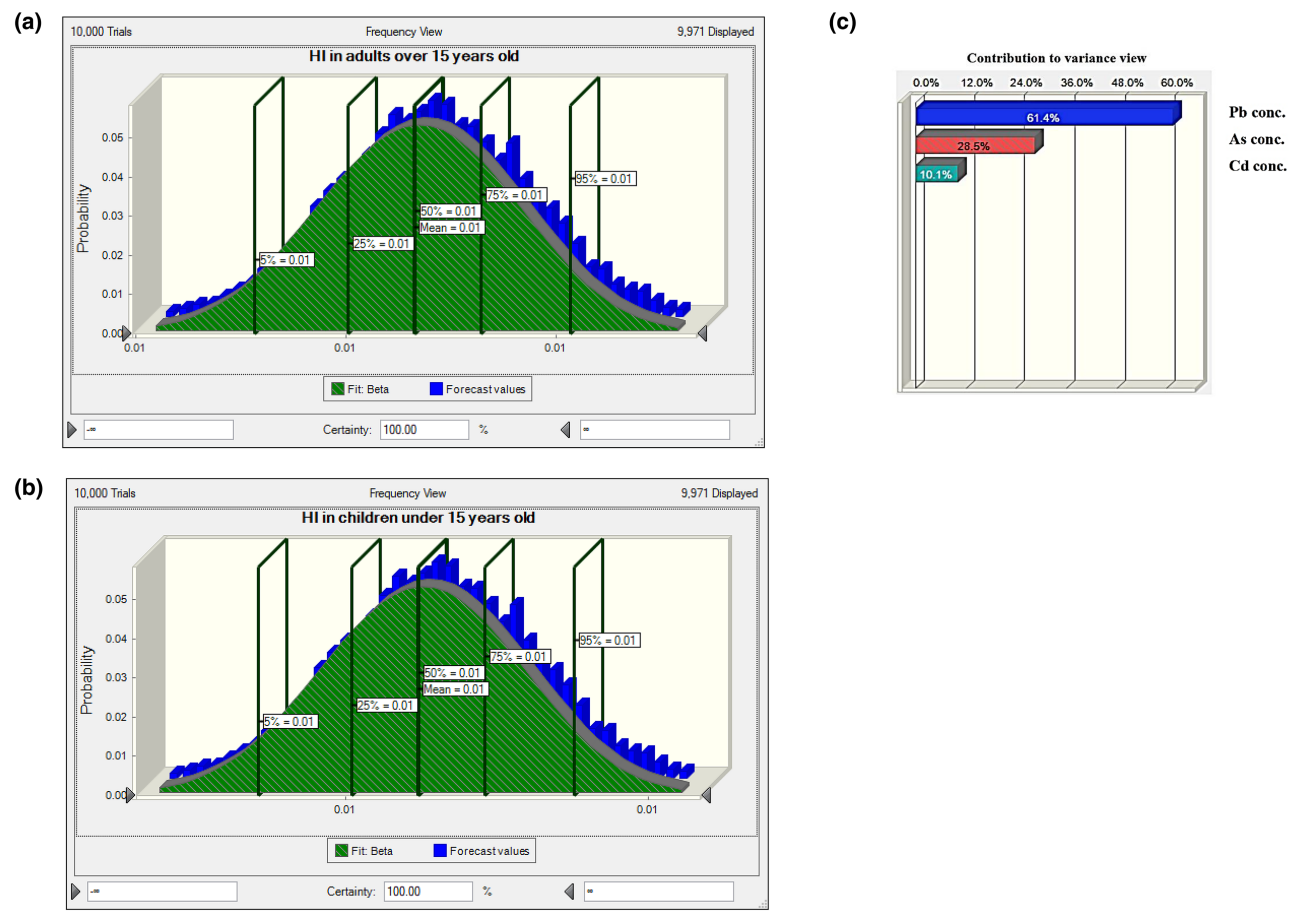
Histogram of Hazard index (HI) for heavy metals ingested by Iranian black tea infusion: (a) in adults, (b) in children, (c) sensitivity chart of HI histogram in both groups.

Considering the wide distribution of heavy metals in the environment, contamination of plant sources with heavy metals is inevitable. Therefore, their intake by human should be considered to evaluate their possible role in the incidence of associated diseases. Other than the likelihood and level of contamination, the frequency of consumption of contaminated foods in individuals has a significant effect on pathogenicity. It is the basis of risk assessment approach to evaluate the burden of contaminants in the development of diseases. Indeed, intake of heavy metals is more important for staple foods that are frequently consumed by individuals. Concerning the high consumption of tea infusion in Asia especially in Iran, we evaluated the noncarcinogenic risk arising from tea infusion by HQ index. To better estimate the risk, we used the Monte Carlo approach by conducting 10,000 iterations in simulation to reduce the uncertainties in calculations. Data are presented for Iranian and foreign tea infusions, separately (Table 5). According to Equation 2 (mentioned above), HQ is the ratio of EDI of a chemical to its RfD. There is no concern when HQ is lower than 1. As seen in Table 5, HQ in both age groups consuming both Iranian and foreign tea infusions was much less than 1. Moreover, HQ was negligible for arsenic and cadmium intake in Iranian tea‐consuming individuals. Our results are in agreement with the findings of SeyyediBidgoli et al. (2022) who assessed the risk of heavy metals in tea infusion in Iran in 2019, although higher HQ for cadmium and lead was reported in their study that is of concern when considering HI. As illustrated in Figures 1a,b and 2a,b, HI in adults and children was lower than 1 for both foreign and Iranian tea consumers. It means that the sum of possible adverse effects of all three heavy metals (arsenic, cadmium, and lead) in all situations was not high enough to induce serious concern in children and adults (HI <1). In comparison, HI of 1.601 was reported in the study of SeyyediBidgoli et al. which was of great concern. They investigated the risk of several heavy metals other than those evaluated in our work. However, the highest value of HQ was calculated for cadmium which is inconsistent with our results. As depicted in Figures 1c and 2c, arsenic and lead had the highest impact on HI in foreign and Iranian tea consumers, respectively.

**FIGURE 3:**
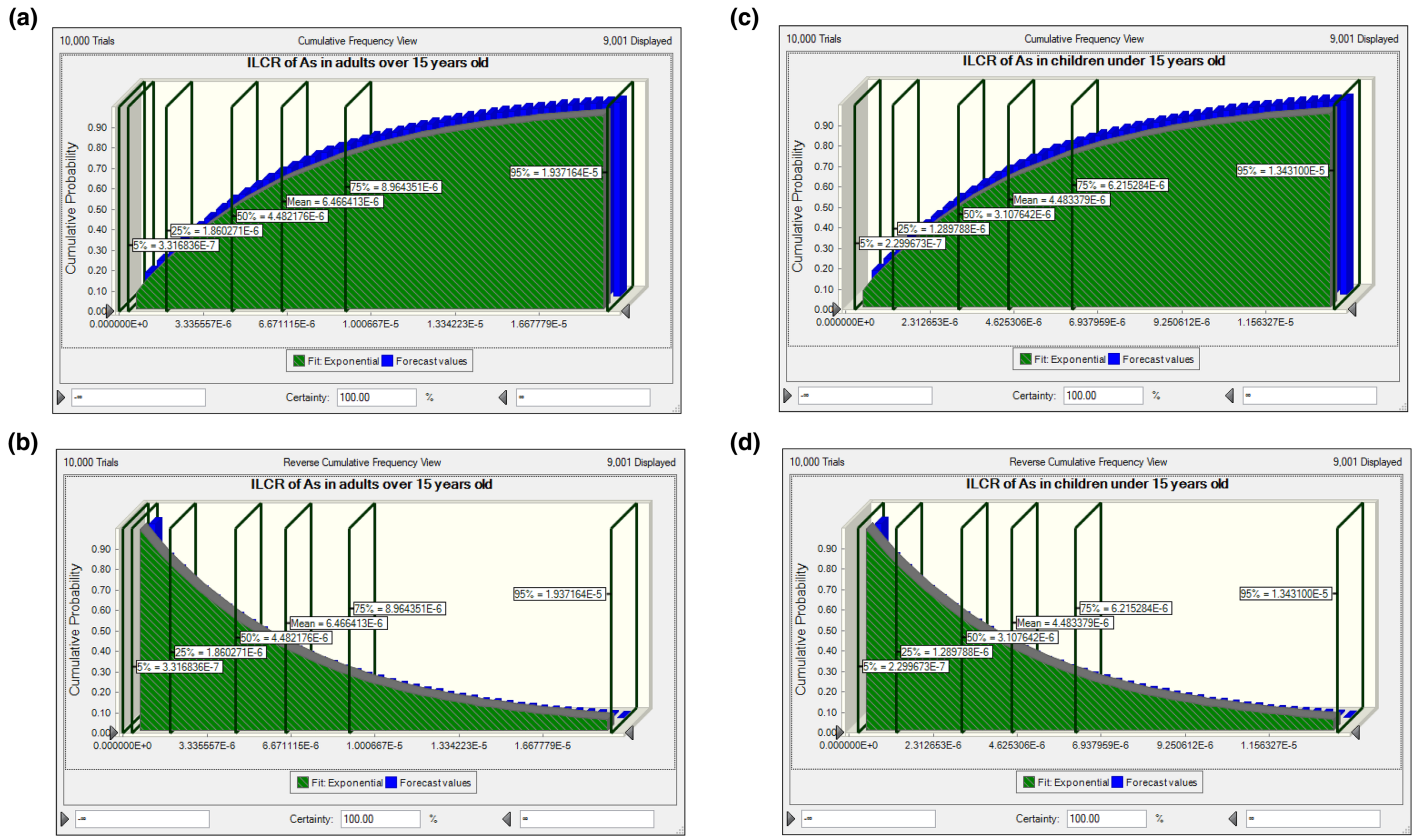
ILCR histogram of arsenic in foreign black tea‐consuming individuals: (a) cumulative frequency graph in adults, (b) reverse cumulative frequency graph in adults, (c) cumulative frequency graph in children, (d) reverse cumulative frequency graph in children.

Carcinogenic risk of arsenic and lead was evaluated with ILCR formula (Table 5). Because no CSF was introduced for cadmium, no evaluation of carcinogenicity was done for this heavy metal. As shown in the table, ILCR of arsenic and lead for both children and adults consuming Iranian black tea infusion was lower than the acceptable range introduced by US EPA (<10–6) (US Environmental Protection Agency, 2001). It was also consistent for lead in both age groups consuming foreign tea infusion. In comparison, ILCR of arsenic in foreign tea‐consuming individuals was within the range of 10^−6^–10^−4^ introduced by US EPA (US Environmental Protection Agency, 2001). Therefore, we referred to its cumulative probability distribution to investigate the location of percentiles in the graph. Figure 3 shows distribution of cumulative frequency for arsenic ILCR in both age groups. As observed in Figure 3a,c, most parts of the histograms are placed at regions below 10^−5^ ILCR (i.e., the cut‐off point introduced by the World Health Organization (Bostrom et al., 2002; Gong et al., 2017)) for both adults and children. It is clearer in the reverse cumulative frequency graphs (Figure 3b,d). However, the risk of arsenic intake in adults is more concerning than in children because more area is located at the right side of 10^−5^ borderline in the histogram of adults compared to children (Figure 3a,b). Nonetheless, there is no serious concern about arsenic intake in each group because both ILCRs were lower than 10^−4^ which is considered a critical point by the US EPA.

Our results showed no safety concern in both children and adults younger and older than 15 years old with respect to intake of arsenic, cadmium, and lead by black tea infusion. We assumed a similar concentration of tea infusion for both age groups, while children drink weaker tea infusion compared to adults. It even provides a wider margin of safety for children consuming tea infusion. However, due to accumulation of heavy metals in vital organs and their long half‐life that is approximately 4 days for arsenic (Hughes, 2006), 1–14 years for cadmium (Suwazono et al., 2009), and 35–40 days for lead (Kumar et al., 2020), assessment of their risk in the consumers should be strictly monitored. In line with the concerns arising from other sources of intake and bioaccumulation of heavy metals, the results reported by Niknejad et al. about risk assessment of heavy metals ingested by rice and water in north of Iran were interested (Niknejad et al., 2023; Niknejad et al., 2024). Accordingly, there was no serious concern about the separate intake of heavy metals from rice and water, but the total intake of heavy metals from all potential sources could be a serious concern, especially in the susceptible groups. Furthermore, we just assessed the risk of three heavy metals by tea consumption, and other routes (i.e., skin and inhalation) have not been investigated.

## 4 CONCLUSION

Heavy metals are widely distributed on the earth and enter the body by contamination of plants and animal foods. These chemicals may pose humans with risk of several cancers and other chronic diseases. Probabilistic risk assessment is a useful approach in the estimation of the risks arising from a chemical that is dangerous to health. In this study, Monte Carlo simulation was used to determine the impact of heavy metals in tea leaves on the pathogenicity of Iranian population. Our evaluation revealed that there is no concern about the noncarcinogenic risk arising from heavy metals in tea leaves marketed in Iran. In this regard, HQ of arsenic and cadmium in Iranian tea consumers was negligible. In addition, HQ was much less than the critical point of 1 for all three heavy metals in foreign tea consumers and for lead in those consuming Iranian tea infusion. With respect to carcinogenicity, ILCR of heavy metals was equal and less than 10^−6^ except for arsenic in foreign tea consumers which was within the range of 10^−6^–10^−4^ determined by US EPA. Therefore, we investigated the cumulative frequency distribution of arsenic ILCR. As a result, the highest area of distribution was related to ILCR lower than 10^−5^ (possibility of <1 cancer in 100,000 individuals). Importantly, the transfer rate of heavy metals from tea leaves to infusion was low for arsenic, cadmium, and lead. It might be due to the low infusion time (10 min) used in our study. Nonetheless, there was no serious concern about heavy metal pathogenicity by consumption of tea infusion in Iran in both children and adults. However, due to bioaccumulation of heavy metals and their long half‐life in vital systems, assessment of their risk through consumption of foods and beverages prone to heavy metal accumulation is strongly recommended.

## AUTHOR CONTRIBUTIONS

**Fatemeh Esfarjani:** Conceptualization (equal); methodology (equal); project administration (equal); writing – review and editing (equal). **Mohammad Rouzbahani:** Formal analysis (equal); methodology (equal). **Seyed Ehsan Beladian Behbahan:** Conceptualization (equal); investigation (equal); project administration (equal). **Narges Shahbazpour:** Data curation (equal); formal analysis (equal). **Masoumeh Moslemi:** Data curation (equal); formal analysis (equal); writing – original draft (lead); writing – review and editing (lead). **Abdol‐Samad Abedi:** Conceptualization (lead); data curation (equal); investigation (equal); methodology (equal); project administration (lead); resources (equal); supervision (equal); visualization (equal).

## CONFLICT OF INTEREST STATEMENT

The authors declare that they have no conflicts of interest to disclose.

## ETHICS STATEMENT

The Ethics Committee of the National Nutrition and Food Technology Research Institute, Faculty of Nutrition Science and Food Technology, Shahid Beheshti University of Medical Sciences approved the project by ethics code: IR.SBMU.NNFTRI.REC.1399.076. This work was not done on animals and/or human participants.

## Data Availability

The data will be available upon request from the corresponding authors.

## References

[fsn34452-bib-0001] Abedi, A. S. , Hoseini, H. , Mohammadi‐Nasrabadi, F. , Rostami, N. , & Esfarjani, F. (2023). Consumer health risk assessment of arsenic and mercury in hen eggs through Monte Carlo simulations. BMC Public Health, 23(1), 1–12. 37430238 10.1186/s12889-023-16223-4 PMC10334572

[fsn34452-bib-0002] Atasoy, A. D. , Yesilnacar, M. I. , Yildirim, A. , & Atasoy, A. F. (2019). Nutritional minerals and heavy metals in tea infusions and daily intake of human body. Turkish Journal of Agriculture‐Food Science and Technology, 7(2), 234–239.

[fsn34452-bib-0003] Beya, M. M. , Netzel, M. E. , Sultanbawa, Y. , Smyth, H. , & Hoffman, L. C. (2021). Plant‐based phenolic molecules as natural preservatives in comminuted meats: A review. Antioxidants, 10(2), 263. 33572049 10.3390/antiox10020263 PMC7915777

[fsn34452-bib-0004] Bimonte, V. , Besharat, Z. , Antonioni, A. , Cella, V. , Lenzi, A. , Ferretti, E. , & Migliaccio, S. (2021). The endocrine disruptor cadmium: A new player in the pathophysiology of metabolic diseases. Journal of Endocrinological Investigation, 44, 1363–1377. 33501614 10.1007/s40618-021-01502-x

[fsn34452-bib-0005] Bostrom, C. E. , Gerde, P. , Hanberg, A. , Jernstrom, B. , Johansson, C. , Kyrklund, T. , Rannug, A. , Törnqvist, M. , Victorin, K. , & Westerholm, R. (2002). Cancer risk assessment, indicators, and guidelines for polycyclic aromatic hydrocarbons in the ambient air. Environmental Health Perspectives, 110(suppl 3), 451–488. 12060843 10.1289/ehp.110-1241197 PMC1241197

[fsn34452-bib-0006] Briffa, J. , Sinagra, E. , & Blundell, R. (2020). Heavy metal pollution in the environment and their toxicological effects on humans. Heliyon, 6(9), e04691. 32964150 10.1016/j.heliyon.2020.e04691 PMC7490536

[fsn34452-bib-0007] Ebrahimi, M. , Khalili, N. , Razi, S. , Keshavarz‐Fathi, M. , Khalili, N. , & Rezaei, N. (2020). Effects of lead and cadmium on the immune system and cancer progression. Journal of Environmental Health Science and Engineering, 18, 335–343. 32399244 10.1007/s40201-020-00455-2 PMC7203386

[fsn34452-bib-0008] Food and Agriculture Organization of the United Nations . (2022). International tea market: market situation, prospects and emerging issues. https://www.fao.org/3/cc0238en/cc0238en.pdf

[fsn34452-bib-0009] Forouzanfar, A. (2011). Review of the therapeutic effects of *Camellia sinensis* (green tea) on oral and periodontal health. Journal of Medicinal Plant Research, 5, 5465–5469.

[fsn34452-bib-0010] Girolametti, F. , Annibaldi, A. , Illuminati, S. , Damiani, E. , Carloni, P. , & Truzzi, C. (2023). Essential and potentially toxic elements (PTEs) content in European tea (*Camellia sinensis*) leaves: Risk assessment for consumers. Molecules, 28(9), 3802. 37175212 10.3390/molecules28093802 PMC10179902

[fsn34452-bib-0011] Glicklich, D. , & Frishman, W. H. (2021). The case for cadmium and lead heavy metal screening. The American Journal of the Medical Sciences, 362(4), 344–354. 34048724 10.1016/j.amjms.2021.05.019

[fsn34452-bib-0012] Gong, Y. , Wei, Y. , Cheng, J. , Jiang, T. , Chen, L. , & Xu, B. (2017). Health risk assessment and personal exposure to volatile organic compounds (VOCs) in metro carriages—A case study in Shanghai, China. Science of the Total Environment, 574, 1432–1438. 27535570 10.1016/j.scitotenv.2016.08.072

[fsn34452-bib-0013] Hayat, K. , Iqbal, H. , Malik, U. , Bilal, U. , & Mushtaq, S. (2015). Tea and its consumption: Benefits and risks. Critical Reviews in Food Science and Nutrition, 55(7), 939–954. 24915350 10.1080/10408398.2012.678949

[fsn34452-bib-0014] Homayouni Rad, A. , Karbalaei, F. , Torbati, M. A. , Moslemi, M. , Shahraz, F. , Babadi, R. , & Javadi, M. (2022). Effect of *Hibiscus sabdariffa* and *camellia synensis* extracts on microbial, antioxidant and sensory properties of ice cream. Journal of Food Science and Technology, 59(2), 1–10. 35185188 10.1007/s13197-021-05068-7 PMC8814224

[fsn34452-bib-0015] Hoseini, H. , Abedi, A. S. , Mohammadi‐Nasrabadi, F. , Salmani, Y. , & Esfarjani, F. (2023). Risk assessment of lead and cadmium concentrations in hen's eggs using Monte Carlo simulations. Food Science & Nutrition, 11(6), 2883–2894. 37324917 10.1002/fsn3.3268 PMC10261825

[fsn34452-bib-0016] Hughes, M. F. (2006). Biomarkers of exposure: A case study with inorganic arsenic. Environmental Health Perspectives, 114(11), 1790–1796. 17107869 10.1289/ehp.9058 PMC1665401

[fsn34452-bib-0017] Islami, F. , Pourshams, A. , Nasrollahzadeh, D. , Kamangar, F. , Fahimi, S. , Shakeri, R. , Abedi‐Ardekani, B. , Merat, S. , Vahedi, H. , Semnani, S. , Abnet, C. C. , Brennan, P. , Moller, H. , Saidi, F. , Dawsey, S. M. , Malekzadeh, R. , & Boffetta, P. (2009). Tea drinking habits and oesophageal cancer in a high risk area in northern Iran: Population based case‐control study. BMJ, 338, 1–8. 10.1136/bmj.b929 PMC3269898 19325180

[fsn34452-bib-0018] Karachaliou, C. , Sgourou, A. , Kakkos, S. , & Kalavrouziotis, I. (2022). Arsenic exposure promotes the emergence of cardiovascular diseases. Reviews on Environmental Health, 37(4), 467–486. 34253004 10.1515/reveh-2021-0004

[fsn34452-bib-0019] Karami, M. A. , Fakhri, Y. , Rezania, S. , Alinejad, A. A. , Mohammadi, A. A. , Yousefi, M. , Ghaderpoori, M. , Saghi, M. H. , & Ahmadpour, M. (2019). Non‐carcinogenic health risk assessment due to fluoride exposure from tea consumption in Iran using Monte Carlo simulation. International Journal of Environmental Research and Public Health, 16(21), 4261. 31684036 10.3390/ijerph16214261 PMC6862652

[fsn34452-bib-0020] Karimi, G. , Hasanzadeh, M. , Nili, A. , Khashayarmanesh, Z. , Samiei, Z. , Nazari, F. , & Teimuri, M. (2008). Concentrations and health risk of heavy metals in tea samples marketed in Iran. Pharmacology, 3(1), 164–174.

[fsn34452-bib-0021] Khan, M. I. , Ahmad, M. F. , Ahmad, I. , Ashfaq, F. , Wahab, S. , Alsayegh, A. A. , Kumar, S. , & Hakeem, K. R. (2022). Arsenic exposure through dietary intake and associated health hazards in the Middle East. Nutrients, 14(10), 2136. 35631276 10.3390/nu14102136 PMC9146532

[fsn34452-bib-0022] Kumar, A. , Kumar, A. , Cabral‐Pinto, M. M. S. , Chaturvedi, A. K. , Shabnam, A. A. , Subrahmanyam, G. , et al. (2020). Lead toxicity: Health hazards, influence on food chain, and sustainable remediation approaches. International Journal of Environmental Research and Public Health, 17(7), 2179. 32218253 10.3390/ijerph17072179 PMC7177270

[fsn34452-bib-0023] Lee, J. G. , Hwang, J. Y. , Lee, H. E. , Kim, T. H. , Choi, J. D. , & Gang, G. J. (2019). Effects of food processing methods on migration of heavy metals to food. Applied Biological Chemistry, 62, 1–10.

[fsn34452-bib-0024] Mahmood, T. , Akhtar, N. , & Khan, B. A. (2010). The morphology, characteristics, and medicinal properties of Camellia sinensis tea. Journal of Medicinal Plant Research, 4(19), 2028–2033.

[fsn34452-bib-0025] Nag, R. , & Cummins, E. (2022). Human health risk assessment of lead (Pb) through the environmental‐food pathway. Science of the Total Environment, 810, 151168. 34710405 10.1016/j.scitotenv.2021.151168

[fsn34452-bib-0026] Naghipour, D. , Amouei, A. , Dadashi, M. , & Zazouli, M. A. (2016). Heavy metal content in black tea and their infusions in North of Iran and estimation of possible consumer health risk. Journal of Mazandaran University of Medical Sciences, 26(143), 211–223.

[fsn34452-bib-0027] Niknejad, H. , Esbakian Bandpei, B. , Abedi Sarvestani, R. , Mohseni‐Bandpei, A. , Saeedi, R. , Abtahi, M. , et al. (2024). Probabilistic health risk assessment of heavy metals in rice produced in Mazandaran province, Iran. Journal of Food Composition and Analysis, 128, 106068.

[fsn34452-bib-0028] Niknejad, H. , Saeedi, R. , Hosseini, S. A. , Abedi Sarvestani, R. , Abtahi, M. , Hesami Arani, M. , Babanezhad, E. , & Gholami‐Borujeni, F. (2023). Health risk assessment of heavy metals in drinking water: A case study in western cities of Mazandaran province, Iran. International Journal of Environmental Analytical Chemistry, 1–16.

[fsn34452-bib-0029] Nishijo, M. , Nogawa, K. , Suwazono, Y. , Kido, T. , Sakurai, M. , & Nakagawa, H. (2020). Lifetime cadmium exposure and mortality for renal diseases in residents of the cadmium‐polluted Kakehashi River basin in Japan. Toxics, 8(4), 81. 33019764 10.3390/toxics8040081 PMC7711806

[fsn34452-bib-0030] OEHHA . (2023). Technical Support Document for Cancer Potency Factors 2009. Appendix A: Hot Spots Unit Risk and Cancer Potency Values. https://oehha.ca.gov/media/downloads/crnr/appendixa.pdf

[fsn34452-bib-0031] Parviz, M. , Eshghi, N. , Asadi, S. , Teimoory, H. , & Rezaei, M. (2015). Investigation of heavy metal contents in infusion tea samples of Iran. Toxin Reviews, 34(3), 157–160.

[fsn34452-bib-0032] Peng, A. , Yu, K. , Yu, S. , Li, Y. , Zuo, H. , Li, P. , Li, J. , Huang, J. , Liu, Z. , & Zhao, J. (2023). Aluminum and fluoride stresses altered organic acid and secondary metabolism in tea (*Camellia sinensis*) plants: Influences on plant tolerance, tea quality and safety. International Journal of Molecular Sciences, 24(5), 4640. 36902071 10.3390/ijms24054640 PMC10003434

[fsn34452-bib-0033] Rady, I. , Mohamed, H. , Rady, M. , Siddiqui, I. A. , & Mukhtar, H. (2018). Cancer preventive and therapeutic effects of EGCG, the major polyphenol in green tea. Egyptian Journal of Basic and Applied Sciences, 5(1), 1–23.

[fsn34452-bib-0034] Rezaee, E. , Mirlohi, M. , Hassanzadeh, A. , & Fallah, A. (2016). Factors affecting tea consumption pattern in an urban society in Isfahan, Iran. Journal of Education Health Promotion, 5, 13. 27500166 10.4103/2277-9531.184568 PMC4960767

[fsn34452-bib-0035] SeyyediBidgoli, N. , Mostafaii, G. R. , Akbari, H. , Mohammadzadeh, M. , Hesami Arani, M. , & Miranzadeh, M. B. (2022). Determination of the concentration of heavy metals in infused teas and their assessment of potential health risk in Kashan, Iran. International Journal of Environmental Analytical Chemistry, 102(19), 7673–7683.

[fsn34452-bib-0036] Shin, S. , Lee, J. E. , Loftfield, E. , Shu, X. O. , Abe, S. K. , Rahman, M. S. , Saito, E. , Islam, M. R. , Tsugane, S. , Sawada, N. , Tsuji, I. , Kanemura, S. , Sugawara, Y. , Tomata, Y. , Sadakane, A. , Ozasa, K. , Oze, I. , Ito, H. , Shin, M. H. , … Sinha, R. (2022). Coffee and tea consumption and mortality from all causes, cardiovascular disease and cancer: A pooled analysis of prospective studies from the Asia cohort consortium. International Journal of Epidemiology, 51(2), 626–640. 34468722 10.1093/ije/dyab161 PMC9308394

[fsn34452-bib-0037] Suwazono, Y. , Kido, T. , Nakagawa, H. , Nishijo, M. , Honda, R. , Kobayashi, E. , Dochi, M. , & Nogawa, K. (2009). Biological half‐life of cadmium in the urine of inhabitants after cessation of cadmium exposure. Biomarkers, 14(2), 77–81. 19330585 10.1080/13547500902730698

[fsn34452-bib-0038] Tang, G. Y. , Zhao, C. N. , Xu, X. Y. , Gan, R. Y. , Cao, S. Y. , Liu, Q. , Shang, A. , Mao, Q. Q. , & Li, H. B. (2019). Phytochemical composition and antioxidant capacity of 30 Chinese teas. Antioxidants, 8(6), 180. 31216700 10.3390/antiox8060180 PMC6617242

[fsn34452-bib-0039] Truong, V. L. , & Jeong, W. S. (2021). Cellular defensive mechanisms of tea polyphenols: Structure‐activity relationship. International Journal of Molecular Sciences, 22(17), 9109. 34502017 10.3390/ijms22179109 PMC8430757

[fsn34452-bib-0040] US Environmental Protection Agency . (1989). Cadmium; CASRN 7440‐43‐9.

[fsn34452-bib-0041] US Environmental Protection Agency . (1991). Arsenic, inorganic; CASRN 7440‐38‐2. 40305639

[fsn34452-bib-0042] US Environmental Protection Agency . (2001). Risk Assessment guidance for superfund: Volume III‐part a. Process for conducting probabilistic risk assessment.

[fsn34452-bib-0043] Wang, M. , Bai, Y. , Wang, Z. , Zhang, Z. , Liu, D. , & Lian, X. (2021). Higher tea consumption is associated with decreased risk of small vessel stroke. Clinical Nutrition, 40(3), 1430–1435. 32943239 10.1016/j.clnu.2020.08.039

[fsn34452-bib-0044] Wang, S. T. , Cui, W. Q. , Pan, D. , Jiang, M. , Chang, B. , & Sang, L. X. (2020). Tea polyphenols and their chemopreventive and therapeutic effects on colorectal cancer. World Journal of Gastroenterology, 26(6), 562–597. 32103869 10.3748/wjg.v26.i6.562 PMC7029350

[fsn34452-bib-0045] Wei, Y. , Peng, X. , Wang, X. , & Wang, C. (2023). The heavy metal‐associated isoprenylated plant protein (HIPP) gene family plays a crucial role in cadmium resistance and accumulation in the tea plant (*Camellia sinensis* L.). Ecotoxicology and Environmental Safety, 260, 115077. 37257351 10.1016/j.ecoenv.2023.115077

[fsn34452-bib-0046] Xi, S. (2022). Partial effect of EGCG, Theanine and tea polysaccharide. In International conference on green environmental materials and food engineering (pp. 172–181). Francis Academic Press.

